# Therapeutic Manuka Honey: No Longer So Alternative

**DOI:** 10.3389/fmicb.2016.00569

**Published:** 2016-04-20

**Authors:** Dee A. Carter, Shona E. Blair, Nural N. Cokcetin, Daniel Bouzo, Peter Brooks, Ralf Schothauer, Elizabeth J. Harry

**Affiliations:** ^1^School of Life and Environmental Sciences, University of Sydney, SydneyNSW, Australia; ^2^The ithree institute, University of Technology Sydney, SydneyNSW, Australia; ^3^University of the Sunshine Coast, MaroochydoreQLD, Australia; ^4^Comvita NZ LimitedTe Puke, New Zealand

**Keywords:** manuka honey, antibacterial, *Leptospermum*, methyl glyoxal, natural product

## Abstract

Medicinal honey research is undergoing a substantial renaissance. From a folklore remedy largely dismissed by mainstream medicine as “alternative”, we now see increased interest by scientists, clinical practitioners and the general public in the therapeutic uses of honey. There are a number of drivers of this interest: first, the rise in antibiotic resistance by many bacterial pathogens has prompted interest in developing and using novel antibacterials; second, an increasing number of reliable studies and case reports have demonstrated that certain honeys are very effective wound treatments; third, therapeutic honey commands a premium price, and the honey industry is actively promoting studies that will allow it to capitalize on this; and finally, the very complex and rather unpredictable nature of honey provides an attractive challenge for laboratory scientists. In this paper we review manuka honey research, from observational studies on its antimicrobial effects through to current experimental and mechanistic work that aims to take honey into mainstream medicine. We outline current gaps and remaining controversies in our knowledge of how honey acts, and suggest new studies that could make honey a no longer “alternative” alternative.

## Introduction

Honey has been used as a medicine throughout the history of the human race. One of the most common and persistent therapeutic uses of honey has been as a wound dressing, almost certainly due to its antimicrobial properties. With the advent of highly active antibiotics in the 1960s, honey was dismissed as a “worthless but harmless substance” (Soffer, 1976). However, the current and growing crisis of antibiotic resistance has revived interest in the use of honey, both as an effective agent in its own right and as a therapeutic lead to develop new methods of treatment. Honey is usually derived from the nectar of flowers and produced by bees, most commonly the European honey bee *Apis mellifera*, and is a complex mix of sugars, amino acids, phenolics, and other substances. Honey types derived from different flowering plants vary substantially in their ability to kill bacteria, and this has complicated the literature on honey and made it sometimes difficult to reproduce results across different studies (Allen et al., 1991; Irish et al., 2011). The majority of recent studies investigating the mechanism of action of honey have focused on well-characterized, standardized active manuka honey produced by certain *Leptospermum* species native to New Zealand and Australia, which has been registered as a wound care product with appropriate medical regulatory bodies. Thus, unless otherwise specified, this review will focus on manuka honey.

## Chemical Analyses of Active Manuka Honey

Professor Peter Molan of Waikato University, New Zealand, was the first to report the unusual activity of manuka honey and began testing its action against a wide range of different bacterial species in the mid 1980s. However, while it was clear that even low concentrations of manuka honey killed bacterial pathogens, the specific active ingredient responsible for this remained elusive for many years. High sugar and low pH make honey inhibitory to microbial growth, but activity remains when these are diluted to negligible levels. Many different types of honey also produce hydrogen peroxide when glucose oxidase, which is derived from the honey bee, reacts with glucose and water. However, in manuka honey hydrogen peroxide production is relatively low and can be neutralized by catalase, yet activity still remains. The cause of this remaining activity, dubbed “non-peroxide activity” or NPA, was finally revealed in 2008, when two laboratories independently identified methyl glyoxal (MGO) in manuka honey (Adams et al., 2008; Mavric et al., 2008). MGO results from the spontaneous dehydration of its precursor dihydroxyacetone (DHA), a naturally occurring phytochemical found in the nectar of flowers of *Leptospermum scoparium, Leptospermum polygalifolium*, and some related *Leptospermum* species native to New Zealand and Australia (Adams et al., 2009; Williams et al., 2014; Norton et al., 2015). MGO can react relatively non-specifically with macromolecules such as DNA, RNA and proteins (Adams et al., 2008; Mavric et al., 2008; Majtan et al., 2014b), and could theoretically be toxic to mammalian cells (Kalapos, 2008). However, there is no evidence of damage to host cells when manuka honey is either consumed orally or used as a wound dressing; indeed honey appears to stimulate healing and reduce scarring when applied to wounds (Biglari et al., 2013; Majtan, 2014; Dart et al., 2015). How it exerts this apparently selective toxicity to bacterial cells is not known.

High levels of MGO or hydrogen peroxide usually produce the most active honey, however, the correlation is not always perfect suggesting other components of honey may modulate activity (Molan, 2008; Kwakman et al., 2011; Chen et al., 2012; Lu et al., 2013). Bee defensin-1, an antimicrobial bee-derived peptide is responsible for activity in Revamil honey, an active honey produced from an undisclosed source, but this appears to be structurally modified and inactive in manuka honey (Kwakman et al., 2011; Majtan et al., 2012). The level of leptosin, a glycoside found exclusively in *Leptospermum* honey, correlates with potency and may modulate the antimicrobial activity of manuka honey (Kato et al., 2012). Similarly, various phenolic compounds with potential antimicrobial activity can be present, particularly in darker colored honeys, and although these occur at levels that are unlikely to be inhibitory on their own they may synergize with one another or other components of honey to produce or alter activity (Estevinho et al., 2008; Stephens et al., 2010). Phenolics can also act as antioxidants and may be responsible for anti-inflammatory and wound-healing properties of honey (Stephens et al., 2010). It should be noted that not all *Leptospermum* species produce active honey, and even within *L. scoparium* and *L. polygalifolium* honey MGO levels can range from ∼100 to >1200 ppm (Windsor et al., 2012). A survey of Australian honey activity found honey sourced from *Leptospermum* plants growing around the New South Wales–Queensland border was particularly active, but whether this is due to plant, soil, climate or other factors is not known (Irish et al., 2011).

## The Inhibition of Pathogens by Honey

Honey has been tested *in vitro* on a diverse range of pathogens, particularly those that can colonize the skin, wounds and mucosal membranes, where topical honey treatment is possible. To date, *in vitro* assays have found manuka honey can effectively inhibit all problematic bacterial pathogens tested (summarized in **Table 1**). Of particular interest is that clinical isolates with multiple drug resistance (MDR) phenotypes have no reduction in their sensitivity to honey, indicating a broad spectrum of action that is unlike any known antimicrobial (Willix et al., 1992; Blair and Carter, 2005; George and Cutting, 2007; Tan et al., 2009). In addition, attempts to generate honey-resistant strains in the laboratory have not been successful and there have been no reports of clinical isolate with acquired resistance to honey (Blair et al., 2009; Cooper et al., 2010).

**Table 1:** Bacterial species found to be susceptible to therapeutic manuka honey.

Bacterial species^1^	No. isolates^2^	Honey type^3^	MIC (%v/v)^4^	Reference
*Acinetobacter baumannii*	11 (C)	Medihoney^®^ (AUST)	6–8^S^	George and Cutting, 2007
	1 (C)	Medical manuka 1	6^S^	Carnwath et al., 2014
	1 (C)	Medical manuka 2	10^S^	
	1 (C)	Manuka 20+ (UB)	4^S^	
	1 (C)	Manuka 10+ (UB)	12^S^	
*Acinetobacter calcoaceticus*	4	Medihoney^®^ (AUST)	8.1^S^	Blair et al., 2009
*Actinomyces pyogenes*	1 (C)	Manuka (1)	5^S^	Allen and Molan, 1997
*Alcaligenes faecalis*	1 (C)	Manuka 10+ (CNZ)	25	Mundo et al., 2004
*Bacillus cereus*	1 (C)	Manuka 10+ (CNZ)	25	
*Bacillus stearothermophilus*	1 (C)	Manuka 10+ (CNZ)	50	
*Bacillus subtilis*	1	Manuka 10+ (CNZ)	10	Balan et al., 2016
*Burkholderia ambifaria*	4 (C)	Woundcare 18+ (CUK/NZ)	4.5	Jenkins et al., 2015b
*Burkholderia anthina*	4 (C)	Woundcare 18+ (CUK/NZ)	4.25	
*Burkholderia cenocepacia*	15 (C)	Woundcare 18+ (CUK/NZ)	4.5	
*Burkholderia cepacia*	20 (C)	Manuka (2)	2–5^S^	Cooper et al., 2000
	6 (C)	Woundcare 18+ (CUK/NZ)	5.2	Jenkins et al., 2015b
*Burkholderia cepacia complex*	4 (C)	Woundcare 18+ (CUK/NZ)	4	
*Burkholderia cepacia group K*	3 (C)	Woundcare 18+ (CUK/NZ)	4.3	
*Burkholderia multivorans*	10 (C)	Woundcare 18+ (CUK/NZ)	4.6	
*Burkholderia pyrrocinia*	2 (C)	Woundcare 18+ (CUK/NZ)	5	
*Burkholderia stabilis*	1 (C)	Woundcare 18+ (CUK/NZ)	5	
*Burkholderia vietnamensis*	5 (C)	Woundcare 18+ (CUK/NZ)	4.8	
*Citrobacter freundii*	2	Medihoney^®^ (AUST)	9.1^S^	Blair et al., 2009
*Clostridium difficile*	3 (2C)	Woundcare 18+ (CNZ)	6.25	Hammond and Donkor, 2013
*Enterobacter aerogenes*	1	Medihoney^®^ (AUST)	13.8^S^	Blair et al., 2009
	1 (C)	Manuka 16+ (SG)	11.9^S^	Lin et al., 2011
*Enterobacter agglomerans*	1	Medihoney^®^ (AUST)	7^S^	Blair et al., 2009
*Enterobacter clocae*	6 (C)	Medihoney^®^ (AUST)	6	George and Cutting, 2007
	18	Medihoney^®^ (AUST)	11.8^S^	Blair et al., 2009
	1 (C)	Manuka 16+ (SG)	10.65^S^	Lin et al., 2011
ESBL producing *Enterobacter clocae*	1	Manuka 16+ (SG)	5.9^S^	
ESBL producing *Enterobacter* sp.	1	Manuka 16+ (SG)	5.9^S^	
*Enterococcus faecalis*	3 (C)	Medihoney^®^ (AUST)	6–8	George and Cutting, 2007
	1 (C)	Medical manuka 1	8^S^	Carnwath et al., 2014
	1 (C)	Medical manuka 2	12^S^	
	1 (C)	Manuka 20+ (UB)	6^S^	
	1 (C)	Manuka 10+ (UB)	10^S^	
*Enterococcus* sp.	7 (C)	Manuka (3)	4.7–5^S^	Cooper et al., 2002b
	3 (C)	Medihoney^®^ (AUST)	6	George and Cutting, 2007
VRE	20	Manuka (3)	3.8–5^S^	Cooper et al., 2002b
	20 (C)	Medihoney^®^ (AUST)	6–8	George and Cutting, 2007
*Escherichia coli*	10 (C)	Medihoney^®^ (AUST)	6–8	
	1	Medical manuka 3	5^S^	Wilkinson and Cavanagh, 2005
	1	Medihoney	2.5^S^	
	1	Manuka 10+ (CNZ)	20	Balan et al., 2016
	9	Medihoney^®^ (AUST)	7.5^S^	Blair et al., 2009
	1 (C)	Medical manuka 1	6^S^	Carnwath et al., 2014
	1 (C)	Medical manuka 2	10^S^	
	1 (C)	Manuka 20+ (UB)	4^S^	
	1 (C)	Manuka 10+ (UB)	8^S^	
	1	Manuka 16+ (SG)	6.9^S^	Lin et al., 2011
	1	Manuka 25+ (CNZ)	12.5^S^	Sherlock et al., 2010
	1 (C)	Woundcare 18+ (CUK/NZ)	12.0	Cooper et al., 2010
ESBL producing *Escherichia coli*	3	Manuka 16+ (SG)	4.7–5.5^S^	Lin et al., 2011
*Escherichia coli* 0157:H7	1 (C)	Manuka 10+ (CNZ)	50	Mundo et al., 2004
	1	Manuka 10+ (CNZ)	10	Balan et al., 2016
*Helicobacter pylori*	28 (C)	Manuka (AHNZ)	10^S^	Osato et al., 1999
	7 (C)	Manuka (2)	5^S^	Al Somal et al., 1994
*Klebsiella pneumonia*	1 (C)	Manuka (1)	10^S^	Allen and Molan, 1997
	12 (C)	Medihoney^®^ (AUST)	6–8	George and Cutting, 2007
	7	Medihoney^®^ (AUST)	13^S^	Blair et al., 2009
*Listeria monocytogenes*	1	Manuka 10+ (CNZ)	20	Balan et al., 2016
*Morganella morganii*	1	Medihoney^®^ (AUST)	7.8^S^	Blair et al., 2009
*Nocardia asteroides*	1 (C)	Manuka (1)	5^S^	Allen and Molan, 1997
*Proteus mirabilis*	1	Manuka (1)	10.8	Willix et al., 1992
*Proteus vulgaris*	1	Manuka 10+ (CNZ)	20	Balan et al., 2016
*Pseudomonas* spp.	20 (C)	Manuka (2)	5.5–8.7	Cooper and Molan, 1999
*Pseudomonas aeruginosa*	17 (C)	Manuka (3)	4–9^S^	Cooper et al., 2002a
	1	Manuka (1)	15.7	Willix et al., 1992
	20 (C)	Medihoney^®^ (AUST)	12–14	George and Cutting, 2007
	1	Medical manuka 3; Medihoney	2.5^S^	Wilkinson and Cavanagh, 2005
	112 (C)	Manuka (AUST UB)	20	Mullai and Menon, 2007
	1	Manuka (AUST UB)	10	
	40 (E)	Manuka (AUST UB)	20	
	56 (C)	Woundcare 18+ (CUK/NZ)	7.3	Jenkins et al., 2015b
	1 (C)	Manuka MGO550	12.5	Anthimidou and Mossialos, 2012
	1 (C)	Medical manuka 1	8^S^	Carnwath et al., 2014
	1 (C)	Medical manuka 2	10^S^	
	1 (C)	Manuka 20+ (UB)	8^S^	
	1 (C)	Manuka 10+ (UB)	10^S^	
	3 (2C)	Medihoney^®^ (CNZ)	10–30%	Kronda et al., 2013
	1	Woundcare 18+ (CUK/NZ)	6	Jenkins and Cooper, 2012
	1	Medical manuka 4	12	Roberts et al., 2012
	2 (1C)	Medihoney^®^ (CNZ)	10–30	Maddocks et al., 2013
	1	Manuka (3)	9.5^S^	Henriques et al., 2011
	1	Manuka 25+ (CNZ)	12.5^S^	Sherlock et al., 2010
	1	Woundcare 18+ (CUK/NZ)	15.7	Cooper et al., 2010
*Salmonella enteritidis*	1	Manuka 10+ (CNZ)	20	Balan et al., 2016
	1	Manuka 16+ (SG)	6.8^S^	Lin et al., 2011
*Salmonella mississippi*	1	Manuka 16+ (SG)	6.8^S^	
*Salmonella typhimurium*	1 (C)	Manuka 10+ (CNZ)	50	Mundo et al., 2004
	1	Manuka 10+ (CNZ)	20	Balan et al., 2016
	2	Manuka 16+ (SG)	6.8–7.5^S^	Lin et al., 2011
*Serratia marcescens*	1	Manuka (1)	9.4	Willix et al., 1992
	1	Medihoney^®^ (AUST)	14.8^S^	Blair et al., 2009
*Shigella flexneri*	1 (C)	Manuka 16+ (SG)	7.58^S^	Lin et al., 2011
*Shigella sonnei*	1	Manuka 10+ (CNZ)	10	Balan et al., 2016
	1 (C)	Manuka 16+ (SG)	6.6 ^S^	Lin et al., 2011
*Staphylococcus aureus*	1 (C)	Manuka (1)	5^S^	Allen and Molan, 1997
	1	Manuka (1)	2.7	Willix et al., 1992
	2	Medihoney^®^ (AUST)	4.4^S^	Blair et al., 2009
	48 (C)	Medihoney^®^ (AUST)	4	George and Cutting, 2007
	1	Manuka 10+ (CNZ)	5	Balan et al., 2016
	1 (C)	Manuka MGO550	6.25	Anthimidou and Mossialos, 2012
	4 (2C)	Manuka (5CNZ)	8	Lu et al., 2013
	1 (C)	Medical manuka 1	6^S^	Carnwath et al., 2014
	1 (C)	Medical manuka 2	10^S^	
	1 (C)	Manuka 20+ (UB)	2^S^	
	1 (C)	Manuka 10+ (UB)	10^S^	
	1	Medihoney^®^ (CNZ)	8	Maddocks et al., 2013
	2 (C)	Medihoney^®^ (CNZ)	6–10	
	2	Manuka (3)	1.2–3.4	Henriques et al., 2010
	1	Woundcare 18+ (CUK/NZ)	3	Cooper et al., 2010
	58 (C)	Manuka (2)	2–3	Cooper et al., 1999
*Staphylococcus aureus* resistant to antibiotics other than methicillin	5 (C)	Medihoney^®^ (AUST)	4.1^S^	Blair et al., 2009
MRSA	18 (C)	Manuka (3)	2.7–3^S^	Cooper et al., 2002b
	13 (C)	Medihoney^®^ (AUST)	4.2^S^	Blair et al., 2009
	1 (C)	Medical manuka 1	4^S^	Carnwath et al., 2014
	1 (C)	Medical manuka 2	10^S^	
	1 (C)	Manuka 20+ (UB)	3^S^	
	1 (C)	Manuka 10+ (UB)	10^S^	
	1	Medihoney^®^ (CNZ); Manuka (CNZ)	8	Müller et al., 2013
	4 (C)	Manuka 25+ (CNZ)	12.5^S^	Sherlock et al., 2010
	1	Manuka 25+ (CNZ)	12.5^S^	
Epidemic MRSA	1	Woundcare 18+ (CUK/NZ)	6	Jenkins and Cooper, 2012
	1	Medihoney^®^ (CNZ)	20	Maddocks et al., 2013
*Staphylococcus* (coagulase negative)	18 (C)	Manuka (4)	2.7–5^S^	French et al., 2005
*Staphylococcus epidermidis*	1 (C)	Woundcare 18+ (CUK/NZ)	7	Cooper et al., 2010
MRSE	1 (C)	Medical manuka 1	4^S^	Carnwath et al., 2014
	1 (C)	Medical manuka 2	10^S^	
	1 (C)	Manuka 20+ (UB)	4^S^	
	1 (C)	Manuka 10+ (UB)	6^S^	
*Staphylococcus equi* subsp. *equi*	1 (C)	Medical manuka 1	6^S^	
	1 (C)	Medical manuka 2	10^S^	
	1 (C)	Manuka 20+ (UB)	4^S^	
	1 (C)	Manuka 10+ (UB)	8^S^	
*Staphylococcus equi* subsp. *zooepidemicus*	1 (C)	Medical manuka 1	6^S^	


	1 (C)	Medical manuka 2	4^S^	
	1 (C)	Manuka 20+ (UB)	10^S^	
	1 (C)	Manuka 10+ (UB)	4^S^	
Staphylococcus sciuri	1 (C)	Medical manuka 1	4%S	
	1 (C)	Medical manuka 2	10^S^	
	1 (C)	Manuka 20+ (UB)	4^S^	
	1 (C)	Manuka 10+ (UB)	6^S^	
*Stenotrophomonas maltophilia*	20 (C)	Manuka 15+ (NN)	7.5–22.5^S^	Majtan et al., 2011
*Streptococcus agalactiae*	1 (C)	Manuka (1)	5^S^	Allen and Molan, 1997
*Streptococcus dysgalactiae*	1 (C)	Manuka (1)	5^S^	
*Streptococcus pyogenes*	1	Medihoney^®^ (CNZ)	20	Maddocks et al., 2012
	2 (1C)	Medihoney^®^ (CNZ)	20	
*Streptococcus uberis*	1 (C)	Manuka (1)	5^S^	Allen and Molan, 1997
*Yersinia enterocolitica*	1	Manuka 16+ (SG)	4.8^S^	Lin et al., 2011


*^1^VRE: Vancomycin-resistant Enterococcus; MRSA: Methicillin-resistantStaphylococcus aureus; MRSE: Methicillin-resistant Staphylococcus epidermidis; ESBL, Extended spectrum β-lactamase.*

*^2^Where source was specified C, clinical isolates; E, environmental isolates.*

*^3^Leptospermum **honeys registered as wound care products:** Medihoney^®^ (CNZ) – A commercial sterile therapeutic honey product, proprietary blend of active Leptospermum honey, Comvita Ltd, New Zealand; Medihoney^®^ (AUST) – A commercial sterile therapeutic honey product, proprietary blend of active Leptospermum honey and honey with high levels of hydrogen peroxide-dependent activity, Medihoney Pty Ltd., Australia; Medical manuka 1 – Commercial wound care manuka honey, unspecified brand 1; Medical manuka 2 – Commercial wound care manuka honey, unspecified brand 2; Medical manuka 3 – Commercial manuka, labeled as antibacterial, unspecified brand 3; Woundcare 18+ (CUK/NZ) – Manukacare/WoundCare 18+ UMF pure Leptospermum scoparium honey, Comvita Ltd, United Kingdom/New Zealand; Medihoney – Therapeutic manuka honey with antibacterial activity, unspecified brand; Medical manuka 4 – Sterile, medical-grade manuka honey from New Zealand (Activon, Advancis Medical, Nottingham, UK). **Standadised, commercially available** Leptospermum **honeys with medicinal claims:** Manuka 10+ (CNZ) – Manuka honey 10+ UMF (Comvita Ltd, New Zealand); Manuka 10+ (UB) – Manuka honey 10+, unspecified brand; Manuka 15+ (NN) – Manuka honey 15+ (Nature’s Nectar, New Zealand); Manuka 16+ (SG) – Manuka honey UMF16+ (SummerGlow Apiaries Ltd., New Zealand); Manuka 20+ (UB) – Manuka honey 20+, unspecified brand; Manuka 25+ (CNZ) – Manuka honey UMF 25+ (Comvita, New Zealand); Manuka MGO550 – Manuka honey MGO 550+/UMF25+, Manuka Health, New Zealand. **Non-standardized** Leptospermum **honeys:** Manuka (1) – Unpasteurised centrifugally extracted monofloral Leptospermum scoparium, New Zealand – activity 13.2% phenol; Manuka (2) – Active manuka honey, with activity equivalent to 13.2% phenol, provided by Professor Peter Molan, University of Waikato, New Zealand; Manuka (3) – Active manuka honey, with activity equivalent to 18% phenol, provided by Professor Peter Molan, University of Waikato, New Zealand; Manuka (4) – Manuka honey with NPA equivalent to 16.8% (w/v) phenol, unspecified source, New Zealand; Manuka (5CNZ) – Active manuka honey, with activity equivalent to 18% phenol, provided Comvita Ltd, New Zealand; Manuka (CNZ) – Manuka honey, from Leptospermum scoparium plantations (Hokianga, New Zealand), provided by Comvita Ltd, New Zealand; Manuka (AHNZ) – Creamed Manuka Honey, Airborne Honey Ltd., Leeston, New Zealand; Manuka (AUST UB) – Manuka honey, unspecified brand, Australia.*

*^4^Minimum inhibitory concentration. As different authors used different methods to determine MIC these may not be directly comparable between studies. S: Organism tested was more susceptible to honey than to an artificial honey solution.*

As well as inhibiting planktonic cells, honey can disperse and kill bacteria living in biofilms. Biofilms are communities of cells that are generally enclosed in a self-produced extracellular matrix and found adhering to surfaces, including wounds, teeth, mucosal surfaces, and implanted devices. Microbes resident in biofilms are protected from antimicrobial agents and they can cause persistent, non-resolving infections. Manuka honey disrupts cellular aggregates (Maddocks et al., 2012; Roberts et al., 2012) and prevents the formation of biofilms by a wide range of problematic pathogens, including *Streptococcus* and *Staphylococcus* species, *Pseudomonas aeruginosa, Escherichia coli, Proteus mirabilis, Enterobacter cloacae, Acinetobacter baumannii*, and *Klebsiella pneumonia* (Maddocks et al., 2012, 2013; Lu et al., 2014; Majtan et al., 2014a; Halstead et al., 2016) Importantly, honey can also disrupt established biofilms and kill resident cells, although a higher concentration is required than for planktonic cells (Okhiria et al., 2009; Maddocks et al., 2013; Lu et al., 2014; Majtan et al., 2014a). Very recently, manuka honey was tested on a multispecies biofilm containing *Staphylococcus aureus, Streptococcus agalactiae, Pseudomonas aeruginosa*, and *Enterococcus faecalis* and was found to reduce viability of all species but *E. faecalis*, which could not be eradicated (Sojka et al., 2016). This has clear clinical implications for using honey on wounds containing biofilms, and understanding how the biofilm enables *E. faecalis*, to survive when it is normally killed by honey is an important and interesting area of future study. MGO appears to be mostly but not fully responsible for the inhibition of biofilms by manuka honey, again highlighting the importance of additional components that modulate activity (Kilty et al., 2011; Lu et al., 2014).

The spectrum of activity of honey toward non-bacterial pathogens is yet to be well established. Recent studies examining the antiviral effect of manuka honey have suggested it has potential for treatment of varicella-zoster virus (the cause of chicken pox and shingles) (Shahzad and Cohrs, 2012) and influenza (Watanabe et al., 2014). Fungal pathogens of the skin, including *Candida albicans* and dermatophyte species are substantially less susceptible than bacteria to manuka honey, but are inhibited by honey with high levels of hydrogen peroxide production (Brady et al., 1996; Irish et al., 2006). Manuka and non-manuka honey have been found to reduce the viability of spores of the microsporidian *Nosema apis*, an important pathogen of bees, but honey could not cure bee infection once this was underway (Malone et al., 2001). There have been very few studies on the use of honey for protozoan or helminth parasites and these have not used honey with well-characterized activity, making it difficult to assess the significance of their findings (Bassam et al., 1997; Nilforoushzadeh et al., 2007; Sajid and Azim, 2012).

## Taking Honey Into Mainstream Medicine: Recent Experimental and Mechanistic Studies Shed Light on How Honey Works

Active manuka honey is widely available as a therapeutic agent and functional food, and most consumers accept it as a holistic, somewhat mysterious product. However, a lack of understanding on how honey kills bacteria and promotes healing limits its acceptance by mainstream medicine where it is still considered “alternative” or “complementary”. The vast majority of research studies on honey to date have been descriptive, however, recent studies are attempting to unravel how honey works and are using mechanistic approaches to determine how it acts at the cellular and the molecular level.

## Ultrastructural Studies of Bacterial Cells and Communities Treated by Honey

Honey can profoundly alter the size and shape of bacterial cells, although the extent of this varies in different bacterial species. Using transmission electron microscopy (TEM), *S. aureus* cultures treated with manuka honey had more cells with completed septa compared to those treated with artificial honey, suggesting cells entered but failed to complete the division stage of the cell cycle, although externally these cells appeared normal by scanning electron microscopy (SEM) (Henriques et al., 2010). More recently, phase-contrast imaging following treatment with a sub-lethal dose of manuka honey found cells of *S. aureus* and *Bacillus subtilis* were significantly smaller and were more likely to have condensed DNA than those growing without honey (Lu et al., 2013). It is difficult to directly compare these studies as they used different amounts of honey and treatment times, but overall the results suggest an uncoupling of growth and cell division, which is often seen in response to nutritional and environmental stresses (Silva-Rocha and de Lorenzo, 2010).

Honey treatment has been reported to cause cultures of the Gram negative species *E. coli* and *P. aeruginosa* to have both abnormally shorter and longer cells (Lu et al., 2013). Interestingly, while *P. aeruginosa* appears to be less susceptible to inhibition by honey than other species, profound cellular changes were seen using TEM and SEM, including furrows and blebs (protrusions of cellular plasma membranes) on the cell surface and a substantial amount of extracellular debris indicative of cell lysis (Henriques et al., 2011). This was verified in a subsequent study using BacLight live-dead fluorescence staining and confocal microscopy, although this also demonstrated that a relatively large number of live cells remained. These studies used 20% (w/v) honey, which was higher that the MBC for their strain of *P. aeruginosa* and substantial inhibition and death would be expected. However, atomic force microscopy (AFM) using sub-bactericidal levels still found substantial cell distortion and blebbing in cells treated with MIC (12%) and half MIC (6%) concentrations, along with substantial cell lysis (Roberts et al., 2012). This apparent degeneration of the *P. aeruginosa* cell was supported by quantitative PCR analysis that showed a 10-fold down-regulation in honey-treated cells of *oprF*, which encodes an outer-membrane porin that is important for structural stability (Jenkins et al., 2015a).

## ‘Omics Analyses Assess the Whole-Cell Response to Inhibition by Honey

The ability to assess whole cell outputs has revolutionized the study of drug-pathogen interactions and has particular value for complex natural products like honey where effects on multiple processes are likely. Microarray and proteomic studies of bacteria exposed to honey suggested an induction of stress-related processes and suppression of protein synthesis (Blair et al., 2009; Jenkins et al., 2011; Packer et al., 2012). While overall this is fairly typical of a response to inhibitory agents, honey produced a unique “signature” of differential expression that included many proteins with hypothetical or unknown functions, suggesting a novel mode of action. Specific genes or proteins found to be down-regulated in ‘omics analyses of *S. aureus* and *E. coli* O157/H7 have functions relating to virulence, quorum sensing and biofilm formation (Lee et al., 2011; Jenkins et al., 2013), and in *P. aeruginosa* there was a down-regulation of proteins involved in flagellation (Roberts et al., 2015). These phenotypes are critical for pathogens to establish and produce invasive infection and indicate that as well as inhibiting growth, honey can reduce the pathogenic potential of infecting bacteria.

**Table 2:** Studies of manuka honey: findings, gaps, and future studies.

Study	Findings to date	Gaps and controversies	Suggested future studies
Chemical analyses	MGO is responsible for most but not all of the antibacterial and anti-biofilm activity in manuka honey; hydrogen peroxide is responsible for most but not all of the activity in non-manuka honeys; leptosin may modulate activity; phenolics can act as antioxidants and promote wound healing.	Constituents that modulate activity, produce synergy between honey and antibiotics and promote wound healing are not known.	Fractionation, purification, and testing of constituents alone and in various combinations.
Pathogen inhibition	Manuka honey inhibits growth of all bacterial pathogens tested, prevents biofilms and can disperse and eradicate pre-formed biofilms.	Few studies on non-bacterial pathogens and on mixed-species biofilms.	Test honey on pathogenic fungi, parasites, and viruses; analyze biofilms produced by consortia of bacteria and yeasts.
‘Omics and systems biology	Treatment with manuka honey results in a unique signature of differential gene expression with down-regulation of stress response and virulence-related genes.	Analyses restricted to differential expression; only single time-points explored; only performed in *E. coli* and *S. aureus*; very little validation.	Contextualize using advanced systems biology tools; assess dynamics of cell response; validate using quantitative PCR and gene deletion/overexpression strains.
Ultrastructure	Vastly different morphological alterations in different bacterial species; suggests *S. aureus* fails to complete cell cycle; *P. aeruginosa* has extensive cell degeneration and lysis.	Few species/strains analyzed to date.	Extend to additional strains and species including mixed-species biofilms and wound biopsies.
Drug interactions	Manuka honey is synergistic and/or enhances activity of a variety of antibiotics, prevents development of resistance and renders resistant strains susceptible; MGO not responsible for synergy.	Only *S. aureus* and MRSA tested to date and substantial differences occur among strains; substance/s causing synergy unknown.	Extend to additional strains and species; test honey fractions to determine compound/s responsible for synergy; determine strain-specific differences in response using ‘omics approaches.
*In vivo* use and clinical trials	Case studies and use of therapeutic manuka honey on wounded animals shows honey can clear infections and promote wound healing.	Robust clinical trials have not been undertaken.	Use data obtained from above to inform treatment and devise clinical trials.


Although still relatively limited in number and scope, the ‘omics analyses conducted to date suggest a complex cellular response to honey with considerable variation in different bacterial species. Advanced systems biology approaches that allow contextualization of the data, and validation studies using quantitative PCR and gene deletion strains, are now required to unravel this complexity, and these may reveal new approaches for drug therapies aimed at inhibiting bacterial growth (Hudson et al., 2012).

## Interactions Between Honey and Conventional Antibiotics

As well as use as a sole agent, there is scope for using honey to augment treatment with conventional antibiotics. This may have particular value when combined with systemic agents that can be delivered to a wound bed via blood circulation while honey is applied topically. Combined treatments can also lower the therapeutic dose of antimicrobial agents and prevent the development of resistance, and in some cases can result in drug synergy, where the combined activity is greater than the sum of the individual activities of each drug partner.

*In vitro* studies combining therapeutically approved manuka honey with antibiotic agents have found a synergistic effect with oxacillin, tetracycline, imipenem and mupirocin against the growth of an MRSA strain (Jenkins and Cooper, 2012). Furthermore, the presence of a sub-inhibitory concentration of honey in combination with oxacillin restored the MRSA strain to oxacillin susceptibility. The authors found down-regulation of *mecR1*, which encodes an MRSA-specific penicillin-binding protein (PBP2A) and suggested this as a mechanism of honey synergy. Strong synergistic activity between manuka honey and rifampicin against multiple *S. aureus* strains, including clinical isolates and MRSA strains, has also been found, and the presence of honey prevented the emergence of rifampicin resistance *in vitro* (Müller et al., 2013). This is of clinical significance as rifampicin penetrates well into tissues and abscesses and is commonly used to treat superficial staphylococcal infections, but rapidly induces resistance and must therefore be used in combination with another agent. An additional finding from this study was that synergy was not due to MGO, as a synthetic honey spiked with MGO was not synergistic with rifampicin.

Understanding how honey affects the action of antimicrobials with well-characterized modes of action may also further our understanding of how honey affects bacterial pathogens. Liu et al. (2014) extended the analysis of synergy to include additional antibiotics and different *S. aureus* and MRSA strains. They suggested that an increased susceptibility to clindamycin and gentamicin might result from the combined effect of down-regulated protein synthesis by honey with inhibition of ribosomes by the antibiotics, while synergy with β-lactam antibiotics could be due to increased oxidative stress caused by both partners. As *S. aureus* and MRSA strains were equally susceptible to the oxacillin-honey combination it appeared that synergy was unlikely to be due to PBP2A down-regulation. In one clinical MRSA isolate, however, there was no increase in sensitivity to clindamycin or gentamicin when honey was present, which is notable as it is the first reported case of a difference in response to honey by MRSA versus *S. aureus*. Investigating this strain-specific difference using transcriptomic or proteomic analyses would be an interesting avenue for future research (Liu et al., 2014).

## Evidence of Efficacy From Animal Studies, Case Reports, and Clinical Trials

Companies that produce and market manuka honey promote high ethical standards and discourage the use of animal models to study infections and wound healing. Manuka honey has, however, been used to treat animals with surgical or accidental wounds, particularly horses, with positive outcomes (Dart et al., 2015; Bischofberger et al., 2016). Case reports using honey for non-healing wounds and ulcers have noted significant improvement with resolution of infection where conventional antibiotics had failed (Regulski, 2008; Smith et al., 2009). However, despite this and the evidence from numerous *in vitro* and *in vivo* models that honey kills problematic wound pathogens, there is a paucity of robust clinical data for manuka honey. There are various reasons for this, including technical difficulties in performing a double-blind placebo-controlled trial on a distinctive substance like honey, ethical considerations, lack of interest by clinical practitioners and cost-versus-benefit to honey companies, whose focus is on natural products and over-the-counter sales where manuka honey and associated dressings already command a premium price. These may change as antibiotic resistance erodes current treatment options and ongoing research highlighting the potential of honey brings it to the attention of medical practitioners.

## Gaps and Emerging Opportunities in the Study of Honey

Great progress has been made recently in our understanding of therapeutic honey, yet its use in clinical medicine remains limited, even when conventional antibiotics are starting to fail. The complexity in honey, which is arguably its greatest strength in killing diverse pathogens and preventing resistance, complicates its study as many factors working together are likely to affect activity. We advocate further mechanistic studies using appropriately registered therapeutic manuka honey, in particular studies that use non-reductionist systems biology approaches, along with detailed chemical and microbiological analyses to elucidate how honey acts at the molecular, cellular and population level, how this can differ in different strains and species of microbial pathogens, and how the host cell responds (**Table 2**). Information gained from these studies can then inform therapy and produce the clinical data required to take honey into mainstream medicine; no longer the alternative therapy used only when all else has failed.

## Author Contributions

This review was written by DC, SB, NNC, DB, and PB and was critically reviewed by RS and EH.

## Conflict of Interest Statement

DC, PB, and EH report grant and non-financial support in the form of manuka honey from Comvita NZ Limited and Capilano Honey Limited; RS is employed by Comvita NZ Limited, which trades in medical grade manuka honey (Medihoney). The rest of the authors declare that the research was conducted in the absence of any commercial or financial relationship that could be construed as a potential conflict of interest.

## Abbreviations

ESBL: extended spectrum β-lactamase
MBC: minimum bactericidal concentration
MGO: methyl glyoxal
MIC: minimum inhibitory concentration
MRSA: methicillin-resistant *Staphylococcus aureus*
MRSE: methicillin-resistant *Staphylococcus epidermis*
NPA: non-peroxide activity
VRE: vancomycin-resistant *Enterococcus*
